# Needle phobia among adult Jordanians: General awareness, prevalence; and exploring microneedles as a promising solution

**DOI:** 10.1371/journal.pone.0291807

**Published:** 2023-09-20

**Authors:** Sharif Abdelghany, Suha Al-Muhaissen, Yazan Al Thaher, Mais Melhem, Majd Fashho, Othman Alfuqaha, Mais Saleh

**Affiliations:** 1 School of Pharmacy, University of Jordan, Amman, Jordan; 2 Faculty of Pharmacy, Philadelphia University, Amman, Jordan; 3 The World Islamic Sciences & Education University, Faculty of Educational Sciences, Amman, Jordan; Hennepin County Medical Center, UNITED STATES

## Abstract

Needle phobia remains a major drawback of conventional injectable medications, leading to avoidance and low adherence among a reasonable portion of patients. Despite this, there is a limited number of studies investigating needle phobia prevalence and symptoms. In this survey, we studied the knowledge and prevalence of needle phobia and its manifestations among 1182 adult Jordanians. Moreover, we assessed the feasibility of microneedles delivery systems as an alternative approach to conventional injectable methods. The results revealed that 28.5% of the participants identified themselves with needle phobia, with a notably higher prevalence among females compared to males (p-value < 0.001). The overall prevalence of needle phobia based on its measured manifestations was found to be 27.4%. The survey also found that 68% of the population were unfamiliar with the concept of microneedles despite the reasonable proportion of the population who were aware of the disadvantages of conventional injectable medications. Furthermore, the survey identified four significant predictors of needle phobia through hierarchical linear regression analysis. Gender, occupation, and negative past experiences with needle injections accounted for 3%, 1%, and 1% of the variance in needle phobia, respectively. In addition, the participants’ preference for microneedles over conventional injectables medications appeared as another significant predictor, contributing 5% of the variance. Overall, the model explained 10% of the variance in needle phobia. Collectively, this study provides an insight into needle phobia prevalence and manifestations in Jordan, while also exploring microneedles as an alternative drug delivery system for patients with needle phobia.

## Introduction

Patients dealing with diabetes and other chronic conditions rely on hypodermic needles as part of their daily routine. Nonetheless, the apprehension towards needles presents a barrier in healthcare, potentially leading to reluctance or avoidance of necessary medical treatment [1]. Needle phobia, an intense form of apprehension towards needles, gives rise to a range of physical, mental, and behavioral symptoms and expressions. Therefore, gaining insights into the prevalence, and related symptoms of needle phobia would be beneficial in enhancing treatment programs and patient compliance. This valuable information would guide healthcare professionals in creating a more comfortable environment for patients and providing education to address misconceptions linked to their fears [2, 3]. Also, understanding the drawbacks of conventional needles can aid in exploring alternative drug delivery systems that can mitigate these drawbacks.

Microneedles are micron-sized noninvasive transdermal delivery systems that gained a great interest in the medical field due to their ability to painlessly perforate the skin and enhance the delivery of the incorporated drug. Therefore, this advanced transdermal delivery system has a great promise in combating needle phobia [4, 5]. Numerous investigations have been conducted on the utilization of microneedles within the medical field, aiming at enhancing transdermal drug delivery. These studies demonstrated enhanced safety, as they decreased the likelihood of needle-stick injuries and improved the effectiveness of microneedles in comparison to topical application [6, 7].

In this survey-based research, we investigated the prevalence of needle phobia among adult Jordanians. Additionally, we evaluated the familiarity of the Jordanian population with needle phobia, their general awareness of the drawbacks linked to conventional needle administration, and explored general phobia levels, general distress, as well as mental, physical, and behavioral symptoms associated with injections.

Furthermore, our investigation extended to probing the general population’s understanding of and potential reception to employing microneedles as a viable alternative to conventional injections. We explored the feasibility of employing microneedles as a pain-free alternative for various injectable dosage forms, encompassing intravenous (IV), intramuscular (IM), and subcutaneous (SC) injections.

## Methodology

### Study design

A descriptive cross-sectional design was used to assess the prevalence of needle phobia among Jordanians, and to explore microneedles as a possible alternative to conventional parenteral administrations. This study followed the Strengthening the Reporting of Observational studies in Epidemiology (STROBE) reporting guidelines.

### Study population and recruitment criteria

The survey was sent to a total of 1225 Jordanians, adults (age ≥ 18), who live in Jordan or abroad. The study included a compulsory Yes/No question for participation, positioned after the study’s title, purpose, and the principal investigator’s contact information, but prior to the survey questions. Consequently, completing the questionnaire was a voluntary decision. Respondents who answered "No" to this question and/or were under 18 years old were subsequently excluded. Participants who answered contradictory answers on two synonymous questions regarding their experience of needle phobia were considered aberrant responses and were excluded from the study. Participants who answered the questionnaire were informed that the questionnaire is voluntary and that they can abandon the study at any time with no consequences.

### Settings

The administration of this questionnaire was based online on Google forms. The questionnaire was distributed on WhatsApp and Facebook between March and May 2021 to participants from different area in Jordan. The link of Google form was sent to Facebook and WhatsApp groups that allows only Jordanians to join. Administration of responses to this survey was anonymous with a limited one response per Google account. Two senior pharmacy students helped in distributing the survey to online groups from various categories and various areas in Jordan. The questionnaire was supplemented with instructions and the participants were advised to contact the principal investigator in case of any assistance and/or uncertainty to ensure reliability in the administration of the questionnaire.

### Questionnaire structure

The questionnaire was constructed based on information collected from review of published relevant literature [8–10]. This questionnaire consisted of two distinct sections. The first section investigated the general awareness and prevalence of needle phobia among the Jordanian population aged 18 and older. The second part investigated the feasibility of microneedles as an alternative delivery system to conventional drug delivery systems. The first part consisted of sample demographic factors including age, gender, occupation, and place of residence. Additionally, it examined the collective understanding and familiarity of the populace regarding needle phobia and its prevalence. The first part of the survey also assessed the general awareness within the Jordanian population regarding the drawbacks of needle injections, the source of the populace’s knowledge concerning needle phobia, and the real occurrence of needle phobia along with its associated symptoms among the Jordanian demographic. The second part investigated the feasibility of utilizing microneedles as an alternative to conventional drug delivery systems. In this section, a paragraph about the concept of microneedles as a potential drug delivery system was introduced to the participants. Subsequently, the next subsections studied the familiarity of the general population with microneedles and their advantages in solving drawbacks associated with conventional parenteral administration. In the last section, the preference of the Jordanian population between microneedles and other routes of administrations comprising oral, intravenous, subcutaneous, intramuscular, and topical was studied.

### Needle phobia scale based on the experience of participants

In this study, we investigated the prevalence of needle phobia among the Jordanian adults as rated by the participants. Participants were asked to rate their needle phobia towards injections from 1 to 10 (1 is the least and 10 is the highest). The assessment of needle phobia encompassed various aspects, including participants’ distress levels associated with injections, overall phobia tendencies (including other phobias) related to injections, psychological manifestations, physiological symptoms connected to injections, as well as apparent behavioral responses towards injections.

Based on review of the relevant literature and previous published scales addressing needle phobia, we modified needle phobia scale to be suitable in our study. Previously published scales showed that needle phobia scales have good reliability and validity [11, 12]. The modified 55-items were assessed using five dimensions, which measured distress, general phobia, mental, physical, and behavioral symptoms related to injection. We asked participants to answer a 5-Point-Likert scale from “Extreme High” to “Not present”. The higher average score levels present the higher needle phobia. Cut-off-point was considered in three levels (Mild, Moderate, and High). Moderate and high levels were considered to have needle phobia. The modified needle phobia scale was available in English and Arabic languages and its psychometric attributes were assessed.

### Validation

The data collection tool was evaluated by a clinical psychiatrist and his comments were carefully studied to amend the questionnaire. Subsequently, the questionnaire was distributed on a pilot scale to 30 participants for feedback, and their comments were carefully examined to refine the survey, The data of the pilot scale individuals were excluded from the data analysis. Content validity tested according to the opinions of six experts specialized in psychiatric, psychology, and related disciplines. Their perspectives were thoroughly studied, and their input were utilized to amend the survey questions. Statistical confirmation of validity and reliability for the manifestations of needle phobia was achieved through the calculation of Cronbach’ Alpha value. Additionally, sampling adequacy was analyzed using Principal Components Analysis (PCA) with Kaiser-Meyer-Olkin (KMO) and Bartlett’s Test of Sphericity. We tested the validity and reliability of needle phobia scale based on the experiences of participants for each manifestation related to needle phobia (rate of distress related to injections, general phobia level, mental symptoms related to injection level, physical symptoms related to injections level, and behavioral symptoms related to injections level).

### Statistical evaluation

The sample size that was included in the data analysis was 1182, and therefore above the required 322 which was calculated based on a prevalence of 0.3, a confidence interval of 95%, and 5% margin of error [13]. Categorical data were presented as frequency and percentages, while continuous data were presented as median and interquartile ranges. Logistic regression was used to evaluate the strength of association of factors that significantly determine needle phobia prevalence. Pearson coefficient (r) was used to evaluate the correlation between needle phobia manifestations and general phobia level. Pearson coefficient was also used to examine the correlation between gender and the prevalence of needle phobia, as self-identified by the participants. Hypothesis testing conducted was two-sided, and a p-value of < 0.05 was considered significant. Data analysis was performed using SPSS® 23.0 (IBM, Chicago, IL).

To determine the predictive value of needle phobia among Jordanians, four models were constructed using hierarchic linear regression analysis. In the first model, we executed analysis on gender, age, and geographical area. In the second model, occupation, educational level, and health status, were added as variables. In the third model, we added three questions related to needle phobia: Q1: Have you ever heard of needle phobia before? Q2: Do you have relatives with needle phobia? Q3: Have you ever had a traumatic experience during injection? Finally, in the fourth model, we included the preference of microneedles over conventional injection routes (Intravenous, Subcutaneous, Intramuscular), as well as oral and topical routes.

### Ethics approval and consent to participate

Consent was taken in written format, and administration of responses to this survey was anonymous, with a limited one response per Google account.

The research was approved by the Scientific Research Committee of the School of Pharmacy at the University of Jordan (SP-UJ) and the Institutional Review Board Committee affiliated with the University of Jordan (IRB: 7/2018).

## Results

### Sample demographic

The number of participants who were included in the data analysis was 1182. The mean age of participants was 25.5 ± 8. Participants were predominately female (74.6%), healthy (92.9%), had bachelor’s degrees (79.1%), and had heard of needle phobia (87.1%). Analysis of participant-rated needle phobia revealed females were statistically higher than males, as evidenced by Pearson correlation analysis (p-value<0.001) [Table 1].

**Table 1:** Sample demographics/characteristics, n = 1182.

		N (%)	Rate of needle phobia^@^ (M±SD)
Age (M±SD)		25.5±8	3.23±1.2
Gender	Female	882 (74.6)	3.52±1.5***
	Male	300 (25.4)	2.38±1.1
Health Status	Healthy	1098 (92.9)	3.19±1.4
	Unhealthy	84 (7.1)	2.91±1.5
	Diabetes Meletus	28 (2.4)	
	Hypertension & Heart Disease	15 (1.3)	
	Other	51 (4.3)	
Place of Residence	North	108 (9.1)	3.67±1.5
	Central	944 (79.9)	3.18±1.5
	South	27 (2.3)	2.64±1.4
	Outside Jordan	103 (8.7)	2.8±1.2
Education	Scholar degree	62 (5.2)	2.95±1.6
	Diploma degree	48 (4.1)	3.65±1.8
	Bachelor’s degree	935 (79.1)	3.07±1.4
	Postgraduate degree	137 (11.6)	3.3±1.3
Occupation			
	Employed	526 (44.5)	3.17±1.5
	Unemployed and students	656 (55.5)	3.07±1.43
Health Care Related Occupation (N = 526)			
	Yes (280, 23.7%)	280 (53.2)	2.98±1.3
	No (246, 20.8%)	246 (46.8)	3.39±1.6

M: Mean, SD: Standard deviation. ^@^as rated by the respondent (from 1–10). *** Pearson’s correlation (female/male comparison), p-value<0.001.

## Participants perception of needle phobia

Our survey revealed that 28.5% of participants self-identified themselves to have needle phobia. Interestingly, 43.5% of the respondents reported experiencing some fear associated with needle injection [Table 2]. This indicates that although some of the population feel fear from injections, they consider themselves with no phobia towards needles. Surprisingly, 12.4% of the participants decided not to take an injection because of fear associated with injection.

**Table 2:** Knowledge & prevalence related to needle phobia, N = 1182.

	N (%)
Disadvantages of conventional needles^@^	
Needle stick injury	780 (66)
Needle phobia	513 (43.4)
The difficulty in preserving some medications including vaccines	427 (36.1)
The need of trained personnel to administer needles	780 (66)
I don’t know	91 (7.7)
Yes, I am aware of needle phobia	1030 (87.1)
Sources of knowledge about needle phobia^@^	
Physician	264 (22.3)
Pharmacist	256 (21.7
Studied about	304 (25.7)
Family members & Friends	681 (57.6)
Social media & TV	546 (46.2)
Books & magazines	73 (6.2)
Others	40 (3.4)
Relatives with known needle phobia	
None	462 (39.1)
Only one relative	262 (22.2)
2 relatives	135 (11.4)
3 relatives	43 (3.6)
More than 3 relatives	64 (5.4)
I don’t know	216 (18.3)
Respondents with traumatic experience while being injected with a syringe needle	495 (41.9)
Respondents experience with symptoms while being injected with a syringe needle^@^	
Never experienced any symptoms	235 (19.9)
Dry mouth	56 (4.7)
Shortness of breath	74 (6.3)
Lightheadedness/dizziness	185 (15.7)
Sweating	125 (10.6)
Nausea	116 (9.8)
Fainting	72 (6.1)
Pain at the site of injection	789 (66.8)
Blue coloration of the site of injection	678 (57.4)
Do you consider yourself to have needle phobia	
No	845 (71.5)
Yes	337 (28.5)
Respondents experience with injections/needles	
Not afraid of injections/needles.	668 (56.5)
Afraid of needles/injections but they had to take the injection and/or never decided not to take injections out of fear.	367 (31.1)
Afraid of injections/needles and decided not to take injections out of fear. The injection was for:	147 (12.4)
Vaccine	91 (7.7)
Blood test	122 (10.3)
Blood donation	88 (7.4)
Injectable medications	99 (8.4)
Local anesthesia for local operations including dental	102 (8.6)

^@^Do not sum up to 100% (Since the participant can select one or multiple items).

As shown in Table 2, a considerable proportion of the participants (43.4%) are acquainted with the correlation between needle phobia and conventional injectables. The source of general Jordanian population knowledge about needle phobia was primarily from family friends (57.6%), and TV and social media (46.2%). Interestingly, a significant proportion (12.9%) were completely ignorant of the concept of needle phobia. Additionally, most of the participants (80.1%) reported they have experienced symptoms associated with needle injection.

### Participants perception of microneedles

Studying the general awareness of the population about microneedles revealed that 68% were unfamiliar with the concept of microneedles [Table 3]. Among the 32% who responded to be familiar with microneedles, approximately half of them knew about microneedles from social media and TV. Although most participants (≥ 78.3%) showed a higher preference for microneedles over conventional parenteral administrations (IV, IM, or SC), they have a similar preference for microneedles to topical form (50%) and less preference (35.5%) of microneedles to oral route of administration.

**Table 3:** Knowledge about microneedles & attitudes towards microneedles administration, N = 1182.

	N (%)
Respondents who have information about microneedles.	378 (32)
Source of knowledge about microneedles.	
Physician	85 (7.2)
Pharmacist	125 (10.6)
Studied about	183 (15.5)
Family members & Friends	52 (4.4)
Social media & TV	189 (16)
Books & magazines	59 (5)
Flaws of traditional injections/needles that microneedles can solve^@^	
Needle stick injury	644 (54.5)
Needle phobia	740 (62.6)
The difficulty in preserving some medications including vaccines	318 (26.9)
The need of trained personnel to administer needles	572 (48.4)
I don’t know	234 (19.8)
The most important drawback of traditional injections/needles that respondent care to be solved:	
Needle stick injury	264 (22.3)
Needle phobia	351 (29.7)
The difficulty in preserving some medications including vaccines	122 (10.3)
The need of trained personnel to administer needles	445 (37.6)
Preference of participants:	
Preference of microneedles over IV injections	995 (84.2)
Preference of microneedles over subcutaneous injections	1081 (91.5)
Preference of microneedles over Oral route	420 (35.5)
Preference of microneedles over IM injections	926 (78.3)
Preference of microneedles over topical route	591 (50)

^@^Do not sum up to 100% (Since the participant can select one or multiple items).

As shown in Table 3, a significant portion of participants, comprising 62.6% and 54.4%, consider needle phobia and needle stick injuries as prominent drawbacks of traditional injections, respectively. Additionally, 19.8% of participants lack awareness regarding the drawbacks associated with conventional injections. A notable trend among most respondents involves addressing critical issues linked to traditional needles, encompassing concerns like needle phobia, needle stick injuries, and the necessity of trained personnel for administration. To illustrate, within the study’s sample of Jordanians, 37.6% are inclined to resolve the need for trained personnel when employing alternatives like microneedles for conventional needles, while 29.7% express a desire to alleviate needle phobia through the adoption of microneedles.

### Development of a manifestation-based needle phobia scale

The validation process outcomes for the newly developed needle phobia scale confirm the validity of the data, characterized by adequate sampling and reliability. This is indicated by a notably strong internal consistency, as evidenced by KMO and Cronbach’s Alpha values exceeding 0.87 and 0.92, respectively, for all measurements. The significance of Bartlett’s test of sphericity (p-value<0.001) further substantiate these findings [Table 4].

**Table 4:** Validity and reliability of rate of distress related to injection, general phobia level, mental symptoms related to injections, physical symptoms related to injection, and behavioral symptoms related to injections.

	Number of Items	M±SD	Median (IQR)	Cronbach’s Alpha	ICC (95%CI)	PCA			
						KMO	Bartelett’s Test of Sphericity	p-value	df
All items	55	101±39.4	89 (70–123)	0.976	0.976 (0.974–0.978)***	0.977	54675.5	0.000	1485
- Rate Of Distress Related To Injections	8	15±7	13 (9–19)	0.923	0.921 (0.914–0.927)***	0.872	7345.2	0.000	28
- General Phobia Level	22	43.2±15.7	41 (31–51)	0.930	0.929 (0.923–0.935)***	0.937	14314.4	0.000	231
-Mental Symptoms Related To Injections Level	8	13.5±6.9	10 (8–17)	0.939	0.939 (0.934–0.944)***	0.946	7232.1	0.000	28
- Physical Symptoms Related To Injections Level	9	16±8.3	12 (9–21)	0.957	0.956 (0.952–0.960)***	0.954	10084.9	0.000	36
- Behavioral Responses Related To Injections Level	8	13.4±7.1	10 (8–17)	0.942	0.941 (0.936–0.946)***	0.938	7792.1	0.000	28

M: Mean, SD: Standard deviation, IQR: Interquartile range, df: Degree of freedom, ***p-value<0.001.

### Needle phobia prevalence based on the developed needle phobia scale

The results in Table 5 suggest that 27.4% of the participants reported overall needle phobia. This is comparable to the results in Table 2, in which 28.5% of the participants identify themselves to be needle phobic. Participants reported getting general phobia levels at the percentage of 28.1%. Distress, Mental, physical, and behavioral symptoms were found to be in the range of 24.3–26.7%.

**Table 5:** Descriptive statistics and frequencies between study variables.

*Variables*	*Levels*	*N (%)*	*Percentage % Moderate & Extreme*	*Overall experience on 5-point Likert scale (M ± SD)*
Distress related to injections	Mild	867 (74)	26.0%	1.83 ± 0.89
	Moderate	272 (22.4)		
	High	43 (3.6)		
General phobia	Mild	850 (71.9)	28.1%	2.03 ± 0.72
	Moderate	296 (25)		
	High	36 (3.1)		
Mental symptoms	Mild	895 (75.7)	24.3%	2.16 ± 0.67
	Moderate	239 (20.2)		
	High	48 (4.1)		
Physical symptoms	Mild	867 (73.3)	26.7%	2.22 ± 0.73
	Moderate	250 (21.1)		
	High	65 (5.6)		
Behavior symptoms	Mild	885 (74.8)	25.2%	2.16 ± 0.72
	Moderate	241 (20.5)		
	High	56 (4.7)		
Overall needle phobia	Mild	859 (72.6)	27.4%	2.07± 0.63
	Moderate	290 (24.5)		
	High	33 (2.9)		

M: Mean. %: Percentage. SD: Standard Deviation.

Studying the relationship between gender or age and the manifestations of needle phobia (rate of distress related to injections, general phobia level, mental symptoms related to injections level, physical symptoms related to injections level, behavioral responses related to injections level) showed a significant correlation between gender and all measures and symptoms (p<0.05). However, a significant correlation was found between age and physical symptoms related to injections (P<0.01), and behavioral responses related to injections (p <0.05) [Table 6].

**Table 6:** Linear regression of the measures and symptoms of needle phobia against age and gender, N = 1182.

	t	
	Gender (Female/male)	Age
All items	6.2*	-
Rate of distress related to injections	4.4*	-
General phobia level	7.5*	-
Mental symptoms related to injections level	4*	-
Physical symptoms related to injections level	4.4*	-1.6**
Behavioral responses related to injections level	4.2*	-1.7*

* p-value<0.05

** p-value<0.01

Hierarchical regression analysis revealed that gender was a significant predictor of needle phobia (model 1) with total variation of 3%. In step 2, occupation was found to be a predictor of needle phobia adding 1% to model 1. In step 3, a past traumatic experience was significant and explained an additional 1% of the variance. Finally, the preference of microneedles over conventional injections (IV, IM, and SC) were significant predictors of needle phobia, adding 5% of the variance. The total model explained 10% of the variance in needle phobia [Table 7]. These results indicate the microneedles is a plausible solution to overcome IV, SC, IM needle phobia.

**Table 7:** Hierarchical regression analysis prediction of needle phobia.

Factors	Model 1			Model 2			Model 3			Model 4		
	B	β	p-value	B	β	p-value	B	β	p-value	B	β	p-value
Step 1												
Gender	0.26	0.18	0.001***	0.26	-0.01	0.001***	0.26	0.18	0.001***	0.25	5.94	0.001***
Age	-0.01	-0.02	0.56	-0.01	0.18	0.81	-0.01	-0.01	0.82	0.00	-0.14	0.89
Geographical area	0.01	0.01	0.73	0.01	0.03	0.71	0.01	0.01	0.79	0.01	0.21	0.84
R^2^	0.03											
F change	13.07											
Step 2												
Occupation				0.04	0.06	0.05*	0.04	0.06	0.04*	0.04	0.05	0.07
Educational level				0.03	-0.02	0.59	-0.01	-0.01	0.74	-0.02	-0.02	0.63
Health status				0.03	0.05	0.09	0.04	0.04	0.16	0.04	0.04	0.16
R^2^				0.04								
F change				7.91								
Step 3												
Q1							-0.03	-0.02	0.60	-0.03	-0.02	0.57
Q2							-0.04	-0.02	0.41	-0.09	-0.05	0.06
Q3							-0.15	-0.12	0.001***	-0.11	-0.08	0.004**
R^2^							0.05					
F change							7.44					
Step 4												
Mic Vs. IV										0.21	0.16	0.001***
Mic Vs. IM										-0.13	-0.09	0.005**
Mic Vs. SC										-0.10	-0.07	0.04*
Mic Vs. Po.										0.01	0.01	0.91
Mic Vs. Top.										0.02	0.02	0.54
R^2^										0.10		
F change										8.96		

B: Unstandardized coefficients. β: Standardized coefficients.

*p-value < 0.05.

**p-value < 0.01.

***p-value < 0.001. Q1: Have you ever heard of needle phobia before? Q2: Do you have relatives with needle phobia? Q3: Have you ever had traumatic experience during injection? Mic Vs. IV: Microneedle Vs. Intravenous injection. Mic Vs SC Injection, Microneedle Vs. Subcutaneous, Mic Vs IM: Intramuscular, Microneedle. Mic Vs. Po: Microneedle Vs. oral route. Mic Vs. Top: Microneedle Vs. Topical route.

**Fig 1:**
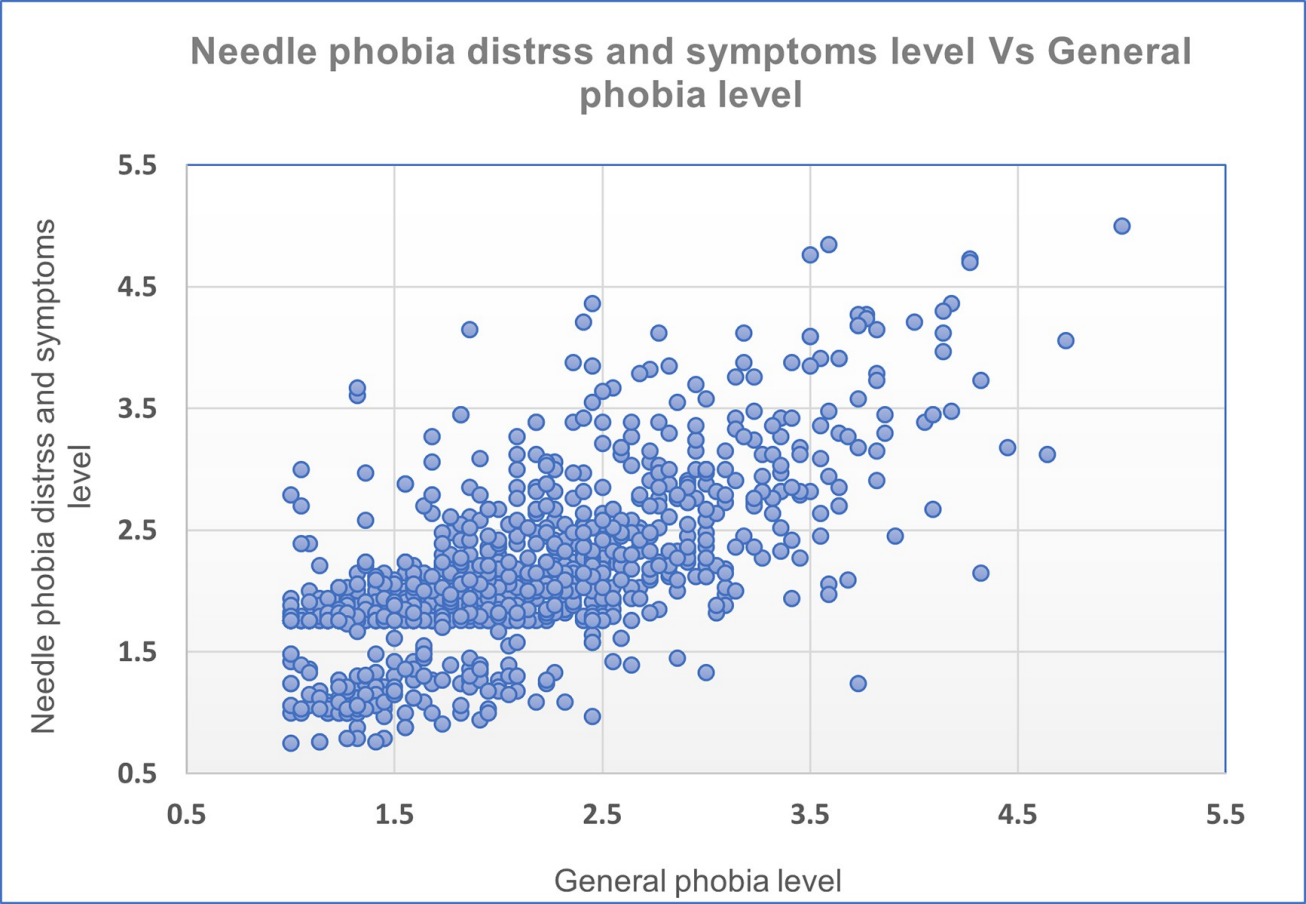
The correlation between needle phobia distress & symptoms, and the measured general phobia level. (Pearson’s coefficient (r) = 0.70, p-value<0.001).

Studying the correlation between manifestations of general phobia (including non-needles related and general anxiety) and needle phobia distress and symptoms for each participant revealed a strong positive correlation, as indicated by Pearson’s correlation of 0.70 (P<0.001) [Fig 1].

## Discussion

The current work described the prevalence of needle phobia in Jordan and the possibility of utilizing microneedles as a potential alternative to conventional parenteral administration.

In our study, the prevalence of needle phobia among adults was found to be 27.4% using a manifestation-based scale, with a significant decrease in the physical symptoms and behavioral responses among participants as their ages increased. The validity, reliability, and sampling adequacy were affirmed by the statistical test we conducted (PCA & Cronbach’s Alpha). Statistical analysis of a previous study that utilized comparable measures for needle phobia (general distress, general phobia level, mental symptoms, physical symptoms, and behavioral symptoms) suggested that these metrics are valid and reliable instruments to assess blood injection phobia in Spanish-speaking individuals [12]. Additionally, a recent systematic review that analyzed 119 research papers from different countries, concluded a needle phobia and fear prevalence of 20% to 30% in adults, with a profound drop in needles phobia to less than 5% in elderly [1]. The summary of these results suggests that needle phobia and fear among adult Jordanians are comparable to other studies. Our study investigated two dimensions: needle fear based on the linked manifestations; and the needle fear based on the participant’s self-report themselves. The similarity of prevalence based on needle fear scale and self-identification (27.4% vs 28.5%) indicates that adult Jordanians were able to identify their fear of needles. Our study showed a strong positive correlation between needle phobia, as a specific phobia, and general phobia level. This is similar to previous results that found a correlation between needle phobia, as a specific phobia, and general non-needle related anxiety [14].

Interestingly, the results that females have greater manifestations linked with needle phobia tend to corroborate with results of studies conducted in India, Mexico, and Saudi Arabia. In contrast, the prevalence of needle fear by gender was different among other countries such as Sweden and Germany, where females showed less needle phobia compared to males. Furthermore, our study revealed that when assessing self-reported fear levels, females displayed a higher prevalence of needle fear compared to males. A recent study among venipuncture participants concluded that females and population with younger age displayed higher tendency to have needle phobia. In that study, psychological and mental symptoms, in addition to pain, were more prevalent among women and in younger age groups [15].

In our study, 12.4% of respondents refused to take injections due to their fear of needles. Recent studies have shown that people who are afraid of needles tend to avoid medical procedures such as blood tests [14], insulin injections for diabetic patients [16], amniocentesis during pregnancy [17], and dental appointments [18]. In a prior investigation involving adult Dutch diabetic individuals who require regular insulin injections, those struggling with self-injection phobia or self-testing phobia exhibited connections to psychological conditions like depression and emotional disturbances. Additionally, they experienced diabetes-related distress, suboptimal treatment compliance, and avoidance tendencies toward diabetes management [19]. This also indicates the association of mental symptoms of needle phobia, general phobia/anxiety level, and needle phobia. In another study among adult US patients in a hospital, 14.8% reported some aversion towards needle injection and blood draw, with only 2.7% have previously avoided injections; furthermore, needle phobic patients reported significantly more manifestations and symptoms compared to patients who responded no fair at all towards needles and injections [15]. In an adolescent sample studying the needle phobia in dental procedures in Norway, avoidance of treatment was recorded at 6.7% for medical treatments and 5.2% for dental treatment [20].

In our study, occupation was a predictor for needle phobia, adding 1% to the needle phobia scale model. This arises from the reality that the professional and social abilities of people can be greatly constrained by their aversion to needles, blood, or injuries [21]. The disorder can also interfere with the ability to perform perinatal care, and limit the choice of potential occupations, particularly those in healthcare professions [22].

In our needle phobia scale model, past traumatic experiences explained 1% of the needle phobia model. This finding was substantiated by a recent comprehensive review that examined the occurrence and treatment of needle phobia among individuals with chronic illnesses across various countries. The review revealed elevated levels of needle fear, with prevalence varying between 17% and 52% among adults who had undergone past or present chemotherapy, ranging from 25% to 47% among adults undergoing peritoneal dialysis or hemodialysis, and reaching up to 80% among diabetic patients [23]. Also, according to direct conditioning pathway of fear acquisition, needle phobia can be developed due to a past traumatic experience [24]. For instance, in a study among children and adolescent participants, 63% have reported that they had experienced a very unpleasant and painful injection [25]. Moreover, in a previous study of 172 participants from Queensland, Australia, participants who had encountered traumatic needle experience in the past exhibited a heightened level of needle phobia in contrast to those with no such traumatic experience [22].

Microneedles are a painless administration that has been proposed to overcome the pain and phobia that are associated with conventional needle injection. In our study, participants preferred microneedles over IV, IM, and SC injections. The acceptability of microneedles and the confidence among volunteers arise from the painless nature and the feasibility of self-administration in the correct way [26]. In a study among Irish pediatrics, the acceptability of using microneedles for the vaccination was tentative by the decision-making parents, and their drive for acceptance was the ability of microneedles to reduce pain, bleeding, and fear [27]. Interestingly, in a recent study, it was shown that utilizing noninvasive medication is the main route to alleviate needle phobia among adult participants [14].

## Recommendations

Contemporary medicine heavily depends on the hypodermic needle for medical tests and drug therapy. Therefore, clinicians must be aware that needle phobia is a prevalent condition that may cause avoidance of necessary medical interventions. Healthcare professionals need to be mindful of the overall discomfort, general fear, psychological and physical manifestations, as well as behavioral reactions linked to needle phobia. In addition, it is highly essential for the health care providers to correctly diagnose patients with needle phobia prior the administration of drugs to prevent untoward sequelae. Revising the patient’s history is essential, with an assessment of the patient’s phobias and anxiety levels. Needle phobia is frequently linked to avoidance behavior and is often responsible for missed appointments. Patients with needle phobia should be taken seriously, and a complete description of the response should be documented. The health care practitioners must exhibit compassion, understanding, and react respectfully to patients with needle phobia.

## Study limitations and weaknesses

A limitation pertinent to this survey is the exclusive Jordanian participation, and therefore this study may need to be conducted in other nations before generalization for global population. Another limitations related to this study is the multiple-choice questionnaire which might compel respondents to answer questions about which they lack sufficient knowledge [28]. We tried to overcome this limitation by adding “I do not know” option, wherever this may arise. Another constraint of this study is its predominant female participation (74.6%) highlighting the apparent reluctance of males to participate in online surveys in our area [29].

## Conclusion

Our findings suggest the significant incidence of needle phobia among adult Jordanians is comparable to the global prevalence. Moreover, needle phobia manifestations and symptoms are evident among adult Jordanians and align with participants’ self-reported prevalence of needle phobia. Given the avoidance of a considerable proportion of adult Jordanians from medical procedures due to their fear of needles, and their inclination towards microneedles over traditional needle-based methods, this presents microneedles as a potential avenue for addressing needle phobia.

## Supporting information

S1 Table A. Rate your distress related to injection, B. General phobia level of the participants, B. General phobia level of the participants, C. Mental symptoms associated with needle injection, D. Level of certain symptoms experienced when receiving an injection, E. Behavioral responses related to needles displayed, N = 1182.
(PDF)
