# Supplementary material for: Needle phobia among adult Jordanians: General awareness, prevalence; and exploring microneedles as a promising solution
Source: PLoS One. 2023 Sep 20;18(9):e0291807. doi: 10.1371/journal.pone.0291807 (PMC10511100; doi:10.1371/journal.pone.0291807)
Supplement: S1 Table — A. Rate your distress related to injection, B. General phobia level of the participants, B. General phobia level of the participants, C. Mental symptoms associated with needle injection, D. Level of certain symptoms experienced when receiving an injection, E. Behavioral responses related to needles displayed, N = 1182. (PDF) [file pone.0291807.s001.pdf]

S1 Table: A. Rate your distress related to injection, B. General phobia level of the participants, B. General phobia level of the participants, C. Mental symptoms associated with needle injection, D. Level of certain symptoms experienced when receiving an injection, E. Behavioral responses related to needles displayed, N=1182.

A. Rate your distress related to injection, N=1182					
	Extreme	High	Moderate	Mild	Not present
Rate your distress related to injection					
Receiving an injection in my arm	54 (4.6)	131 (11.1)	331 (28)	198 (25.2)	368 (31.1)
Holding a needle	27 (2.3)	84 (7.1)	208 (17.6)	249 (21.1)	614 (51.9)
Touching a needle	26 (2.2)	72 (6.1)	192 (16.2)	240 (20.3)	652 (55.2)
Watching someone else having an injection in real life	50 (4.2)	130 (11)	206 (17.4)	252 (21.3)	544 (46)
Watching someone having an injection on TV or online	23 (1.9)	75 (6.3)	164 (13.9)	212 (17.9)	708 (59.9)
Looking at photos of injections	15 (1.3)	54 (4.6)	134 (134)	220 (18.6)	759 (64.2)
Listening to someone talking about having an injection	12 (1)	54 (4.6)	127 (10.7)	234 (19.8)	755 (63.9)
Thinking about having an injection	56 (4.7)	105 (8.9)	185 (15.7)	273 (23.1)	563 (47.6)

B. General phobia level of the participants, N=1182					
	Extreme	High	Moderate	Mild	Not present
Walking on the heights	108 (9.1)	222 (18.8)	357 (30.2)	272 (23)	223(18.9)
Seeing a wounded person bleeding after an accident on the road	101 (8.5)	253 (21.4)	329 (27.8)	230 (19.5)	269 (22.8)
Expressing your opinion in front of your friends or colleagues at work	25 (2.1)	89 (7.5)	242 (20.5)	387 (32.7)	439 (37.2)
Going to the dentist for a medical examination	70 (5.9)	157 (13.3)	255 (21.6)	340 (28.8)	360 (30.5)
Seeing blood on my arm or finger after being punctured with a needle	31 (2.6)	61 (5.2)	116 (9.8)	286 (24.2)	688 (58.2)
When receiving an intravenous injection	73 (6.2)	133 (11.3)	247 (20.9)	296 (25)	433 (36.7)
Cleaning a wound	59 (5)	122 (10.3)	242 (20.5)	314 (26.6)	445 (37.6)
When you want to ask something or express a complaint in public	30 (2.5)	92 (7.8)	236 (20)	350 (29.6)	474 (40.1)
Noticing an advertisement on the correct use of subcutaneous needles	22 (1.9)	44 (3.7)	118 (22.4)	265 (22.4)	733 (62.1)
Seeing a lab tube with blood inside (actually, or on TV)	17 (1.4)	50 (4.2)	105 (8.9)	231 (19.5)	779 (65.9)
Thinking of having to accompany a relative for a blood test or an open wound	41 (3.5)	90 (7.6)	169 (14.3)	265 (22.4)	617 (52.2)
When walking through a dark place	63 (5.3)	102 (8.6)	254 (21.5)	351 (29.7)	412 (34.8)
Receiving local anesthesia	42 (3.6)	90 (7.6)	206 (17.4)	280 (23.7)	564 (47.7)
When I'm in an elevator.	34 (2.9)	61 (5.2)	144 (12.2)	271 (22.9)	672 (56.9)
When thinking that the nurse has to insert a needle into my vein to draw blood out	78 (6.6)	103 (8.7)	186 (15.7)	290 (24.5)	525 (44.4)
Seeing an operation	113 (9.6)	158 (13.4)	299 (25.3)	248 (21)	364 (30.8)
Going to the hospital	35 (3)	73 (6.2)	177 (15)	342 (28.9)	555 (47)
When thinking about the need of local anesthesia for a minor intervention.	55 (4.7)	112 (9.5)	230 (19.5)	320 (27.1)	465 (39.3)

When I'm invited to eat in a restaurant with other people	14 (1.2)	18 (1.5)	116 (9.8)	248 (21)	786 (66.5)
Playing football in a public place	15 (1.3)	13 (1.1)	85 (7.2)	190 (16.1)	879 (74.4)
Riding a bus or train	14 (1.2)	29 (2.5)	103 (8.7)	230 (8.7)	806 (68.2)
When touching a sharp object	23 (1.9)	46 (3.9)	185 (15.7)	349 (29.5)	579 (49)
C. Mental symptoms associated with needle injection, N=1182					
	Extreme	High	Moderate	Mild	Not present
level of certain mental symptoms respondent has prior to receiving injections					
I don't think I can handle the situation	27 (2.3)	64 (5.4)	140 (11.8)	242 (20.5)	709 (60)
I think that something bad will happen to me	25 (2.1)	67 (5.7)	150 (12.7)	294 (24.9)	646 (54.7)
I feel empty minded	22 (1.9)	261 (22.1)	167 (14.1)	261 (22.1)	671 (56.8)
I think others will notice my fear	21 (1.8)	74 (6.3)	166 (14)	246 (20.8)	675 (57.1)
I think I'm going to faint	34 (2.9)	47 (4)	109 (9.2)	216 (18.3)	776 (65.7)
I think I should get out of here before I embarrass myself	25 (2.1)	48 (4.1)	104 (8.8)	177 (15)	828 (70.1)
I think I should've avoided the situation (receiving an injection), because this feeling isn't new	35 (3)	53 (4.5)	125 (10.6)	194 (16.4)	775 (65.6)
I remember past experiences and therefore, I expect the same level of fear	45 (3.8)	86 (7.3)	132 (11.2)	214 (18.1)	705 (59.6)
D. Level of certain symptoms experienced when receiving an injection, N=1182					
	Extreme	High	Moderate	Mild	Not present
Increase in heart rate	54 (4.6)	106 (9)	214 (18.1)	299 (25.3)	509 (43.1)
Increase in sweating	33 (2.8)	61 (5.2)	152 (12.9)	269 (22.8)	667 (56.4)
My muscles start to twitch/spasm	45 (3.8)	82 (6.9)	208 (17.6)	276 (23.4)	571 (48.3)
I get dizzy	38 (3.2)	50 (4.2)	126 (10.7)	222 (18.8)	746 (63.1)
I begin to breath in a fast manner	38 (3.2)	75 (6.3)	170 (14.4)	231 (19.5)	668 (56.5)
My face gets hot	35 (3)	74 (6.3)	182 (15.4)	234 (19.8)	627 (55.6)
I turn pale	31 (2.6)	63 (5.3)	153 (12.9)	226 (19.1)	709 (60)
My stomach gets irritated	38 (3.2)	81 (6.9)	159 (13.5)	250 (21.2)	654 (55.3)
I feel pain in my throat	19 (1.6)	30 (2.5)	92 (7.8)	194 (16.4)	847 (71.7)
E. Behavioral responses related to needles, N=1182					
	Extreme	High	Moderate	Mild	Not present
I avoid receiving injections	56 (4.7)	88 (7.4)	161 (13.6)	238 (20.1)	639 (54.1)
I feel that I can't move while waiting to receive an injection	39 (3.3)	73 (6.2)	131 (11.1)	220 (18.6)	719 (60.8)
My hands/legs shiver while waiting to receive an injection	39 (3.3)	63 (5.3)	152 (12.9)	230 (19.5)	698 (59.1)
I go to get an injection, but I leave the place before my turn comes	12 (1)	24 (2)	73 (6.2)	152 (12.9)	921 (77.9)
I nervously change the way I sit while waiting to receive an injection	47 (4)	98 (8.3)	175 (14.8)	240 (20.3)	622 (52.6)
I can't speak properly and my voice is unbalanced while waiting to receive an injection	34 (2.9)	53 (4.5)	109 (9.2)	196 (16.6)	790 (66.8)
I become speechless and quiet as I wait to receive an injection	31 (2.6)	48 (4.1)	146 (12.4)	208 (17.6)	749 (63.4)
I nervously ask everyone around me questions about receiving an injection	31 (2.6)	57 (4.8)	102 (8.6)	187 (15.8)	805 (68.1)
